# Microleakage Under Orthodontic Brackets Bonded Using a Conventional Adhesive System, Nanocomposites, and Nanoionomers: A Comparative Study

**DOI:** 10.7759/cureus.52537

**Published:** 2024-01-18

**Authors:** Anjali V Rajan, Ratna Parameswaran, Nayeemullah Khan, Sumitra Balaguru

**Affiliations:** 1 Orthodontics and Dentofacial Orthopaedics, Meenakshi Ammal Dental College and Hospital, Chennai, IND

**Keywords:** stereoisomicroscope, nanocomposite, nanoionomer, adhesives, microleakage

## Abstract

Background

Microleakage under orthodontic brackets has a role in early bracket debonding and white spot lesions due to the ingress of oral fluids in the bracket-adhesive-tooth interface. This in vitro study aimed to evaluate and compare the microleakage under orthodontic brackets bonded with the conventional adhesive system, nanocomposites, and nanoionomers.

Materials and methods

Forty-five human premolars were extracted for therapeutic reasons and collected for this study. Teeth were randomly assigned to three groups: Group A: conventional etching with conventional adhesive, Group B: conventional etching with nanocomposite, and Group C: conventional etching with nanoionomer. Stainless steel premolar brackets were used for bonding. After thermocycling, the extracted teeth were submerged in a 0.5% solution of basic fuchsine for 24 hours. They were then cut longitudinally. A stereomicroscope was used to assess microleakage along the occlusal and gingival interfaces.

Results

To compare the microleakage scores between gingival and occlusal aspects within each group, the student's t-test was performed. Analysis of variance (ANOVA) and the post-hoc Tukey test were applied to the data for intergroup comparison of microleakage scores. On comparing the microleakage scores, the gingival side of interfaces depicted higher microleakage than the occlusal side gingival side across all groups, demonstrating statistically significant results (P <0.05).On intergroup comparison, the occlusal bracket adhesive interface and gingival tooth adhesive interface revealed significantly higher microleakage values for Group B followed by Group C with Group A the least. However, there was no significant difference in the microleakage scores between Group B and Group C on intergroup comparison of mean microleakage scores (P <0.05).

Conclusion

The gingival side showed higher microleakage scores than the occlusal side. Nanoionomers showed microleakage values higher than the Transbond XT but lower than nanocomposites. Based on the results of the present study, in terms of microleakage, Transbond XT demonstrated lower microleakage when compared to nanoionomers and nanocomposites.

## Introduction

Microleakage in orthodontics is referred to as the exudation or leakage of fluids between the tooth-adhesive-bracket interfaces, attributed to various etiological factors such as shrinkage due to resin polymerization, enamel and the adhesives' varying thermal expansion, and lack of sufficient adhesion [1]. White spot lesions, well known as the orthodontic cicatrix, are not only seen in areas of teeth that surround the bracket but also beneath the bracket [2]. In congruence with the aforementioned statement, the clinically undetectable anomaly beneath the orthodontic brackets, known as microleakage, is of immeasurable clinical relevance.

In an attempt to overcome the flaws associated with microleakage, the concept of incorporating nanotechnology in resin composite materials and resin-modified glass ionomer cement, gained utmost recognition. In nano-composites, the filler load is increased due to smaller particle dimensions and wider distribution consequently reducing the polymerization shrinkage and improving the mechanical properties [3]. Geraldeli and Perdigao et al. found that when compared to total-etch adhesives, nano-composites had an excellent peripheral seal to enamel and dentine [4]. On the contrary, the study by Shamaa and Hedayati et al. demonstrated higher microleakage scores for nanocomposites than the conventional adhesive system [5,6].

The benefits of resin-modified glass ionomer cement and nanofiller technology have led to the introduction of nanoionomers that avert tooth decay due to their anti-cariogenic and remineralization properties, thus combating microleakage and thereby enamel demineralization in the long run [7]. Upadhyay and Rao et al. found that the degree of microleakage for nanoionomers was less than the conventional and resin-modified glass ionomer cement (GIC) under a standard class V cavity [8]. Uysal et al. have stated that bond strength values of nanoionomers were within the clinically acceptable limits [9].

No research has been published in the literature that evaluates and compares the microleakage scores of conventional adhesives, nanocomposites, and nanoionomers.

This study aimed to compare the microleakage scores of nanoionomers and nanocomposites with conventional adhesives under metal brackets. The null hypothesis is that there was no significant difference in microleakage scores under stainless steel metallic brackets using three different adhesive systems (Transbond XT (3M Company, Saint Paul, Minnesota, United States), nanocomposite, and nanoionomer) at the bracket-adhesive interface and the adhesive-enamel interface.

## Materials and methods

This study commenced after proper review and approval by the institutional ethical committee of Meenakshi Academy of Higher Education and Research (MADC/IRB-XXXI/2019/490). The sample consisted of 45 human premolars that were extracted for therapeutic reasons pertaining to orthodontic treatment. The collected specimens were carefully evaluated for carious and/or non-carious defects, traumatic damage, and developmental defects, and if existent, were excluded. The collected specimens were cleaned and stored in 0.1% thymol solution at room temperature for a minimum of two weeks, to ensure disinfection. The adequately sterilized teeth were then stored in deionized distilled water, before the commencement of the experimental procedures. The sample was randomly divided into 3 equal groups of 15 specimens each. Preliminary to bonding, care was taken to ensure that each tooth was thoroughly debrided and further polished for 10 seconds with a rubber cup, non-fluoridated pumice, and water. The slurry thus formed was washed off via thorough rinsing and drying with an air-water syringe for 10 and 5 seconds, respectively.

The buccal surface of premolars was acid-etched with 37% phosphoric acid gel (3M Dental Products, St. Paul, Minnesota, USA) for 20 seconds. An air-water syringe was used to rinse and dry the teeth for 10 and 5 seconds, respectively, until a frosty white appearance on the enamel was evident. The sample preparation procedures (described above) were equivalent across all groups, i.e., Groups A, B, and C, tested in this study. Following enamel preparation, metal brackets (ORTHO organizers 0.022 ELITE OPTI-MIM SET, CA, USA) were bonded onto each tooth. LED curing unit (3M™ Ortholux™ Luminous Curing Light, State of Minnesota, U.S.) with a 10 mm diameter light tip was used for optimum curing.

The brackets were bonded in the prescribed sequence after acid etching.

Group A (n=15): The samples of this group were bonded with Transbond XT primer (3M Unitek, Monrovia, CA, USA) and conventional light cure adhesive (Transbond XT). After enamel preparation, the primer was applied and thinned with a whiff of air from the air-water syringe and was left uncured after which metallic brackets with conventional adhesive were positioned. The brackets were pressed firmly, with a bracket positioning gauge and excess flash was removed using a hand scaler. The adhesive was light-cured mesially and distally, for 3 seconds, respectively.

Group B (n=15): The teeth in this group were bonded using Filtek Z 350 (3M ESPE, St. Paul, MN, USA). Identical methods of etchant application, primer application (Transbond XT primer), bracket bonding, and curing were followed as in Group A.

Group C (n=15): The specimens belonging to this group were bonded using Ketac Nano Primer (3M ESPE, St. Paul, MN, USA) and nanoionomer (Ketac ™ N100, 3M Espe, St. Paul, MN, USA). After standard tooth preparation, the primer was applied on the enamel surface for 15 seconds and subsequently dispersed onto the surface using a light stream of dry air and later cured. One click of nanoionomer (paste-paste system) was dispensed onto the mixing pad for each bracket and mixed for 20 seconds. The mixed paste was further loaded onto the bracket base with a spatula. Bonding and curing procedures were similar in the aforementioned groups.

Thermocycling of all the samples was performed with a dwell duration of 30 seconds, a transfer time of 10 seconds, and at 5±2°C to 55± 2°C for 1000 cycles, to simulate the effect of percolation.

Microleakage evaluation

Dye penetration was the technique implemented for microleakage evaluation. Before dye penetration, the apices of all specimens were sealed with sticky wax. A double coat application of nail varnish was carried out on all surfaces of teeth, excluding an area of one mm away from the brackets, to intercept the dye penetrating other surfaces. To minimize dehydration, all the specimens were immersed in the water soon after the colored nail varnish was applied and dried.

Each specimen was then immersed in a solution of basic fuchsine standardized to 0.5% at room temperature, for 24 hours. Excess surface dye was brushed off after thorough rinsing with tap water. Teeth were mounted on resin blocks once the specimens were dried.

Using a low-speed diamond disc of dimension 19 mm*0.3 mm (Lemgo, North Rhine-Westphalia, Germany), an axial cut (longitudinally) was made either mesial or distal to each bonded bracket, to increase the measurement accuracy of penetration.

**Figure 1:**
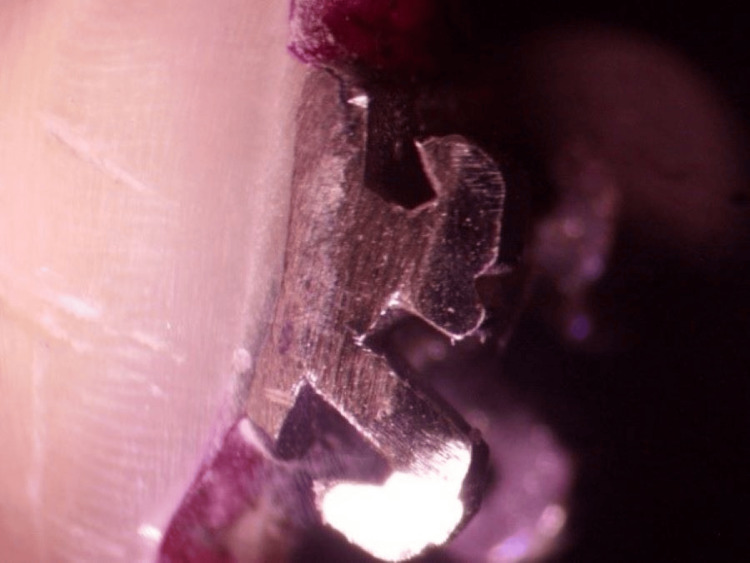
No microleakage under a metal bracket

**Figure 2:**
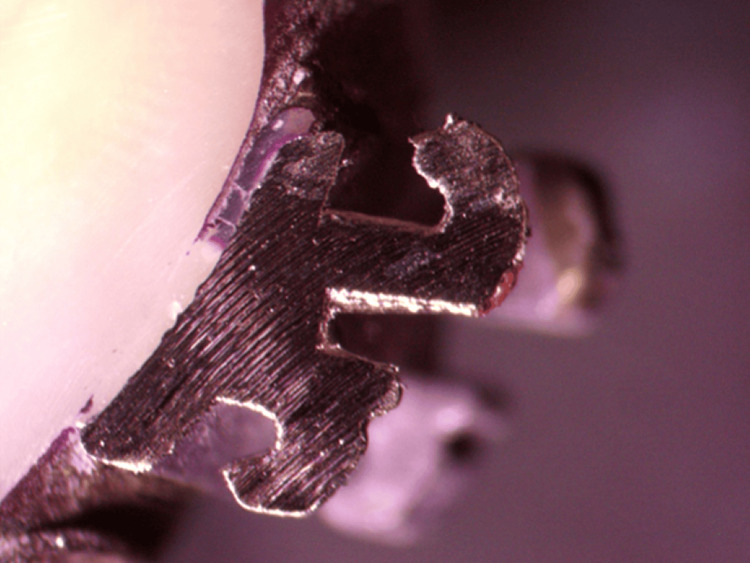
Microleakage under a metal bracket

A stereomicroscope (Lawrence and Mayo LYNX LM 523621, Mumbai, Maharashtra, India ) was used to directly measure and record the microleakage in millimeters using Image J software (V 1.8.0), by a single blinded observer. Adhesive-bracket and enamel-adhesive interfaces were scored from the occlusal and cervical margins of the brackets (Figure 1, Figure 2).

Scoring was made according to the following criteria [10]:

0 = no dye penetration between the bracket-adhesive or the adhesive-enamel interface

1 = dye penetration restricted to 1 mm of the bracket adhesive or adhesive-enamel interface

2 = dye penetration into the inner half (2 mm) of the bracket-adhesive or adhesive-enamel interface

3 = dye penetration into 3 mm of the bracket-adhesive or adhesive enamel interface

The descriptive statistics including the mean and SD values for all three groups were analyzed. The data followed a Gaussian distribution. To compare the microleakage scores between gingival and occlusal aspects within each group, the student's t-test was performed. Analysis of variance (ANOVA) and the posthoc Tukey test were applied to the data for intergroup comparison of microleakage scores. Statistical significance was set at a p-value of <0.05 in the current study. All statistical analysis was performed using IBM SPSS statistics for Windows, version 20.0. Armonk, NY: IBM Corp.

## Results

Measurements were recorded with regard to four interfaces (occlusal tooth adhesive, occlusal bracket adhesive, gingival tooth adhesive, and gingival bracket adhesive interface). The student's t-test revealed statistically significant differences in microleakage scores among the three groups. On comparing the microleakage at occlusal tooth adhesive and gingival tooth adhesive interfaces, there was a significant difference in microleakage scores at the occlusal and gingival tooth adhesive interfaces in Group B (Nanocomposite) and Group C (Nanoionomer). Group A (Transbond XT) on the other hand showed statistically insignificant values between the occlusal and gingival tooth adhesive interface (Table 1).

**Table 1:** Comparison of occlusal tooth adhesive and gingival tooth adhesive interface The level of significance is P <0.05

Sl. No	Groups	Tooth adhesive interface	N	Mean ± SD	Mean difference ± SD	t-value	P-value
1	Group A	Occlusal	15	0.19±0.38	-0.24±0.85	-1.10	0.288
		Gingival	15	0.43±0.7			
2	Group B	Occlusal	15	0.41±0.41	-0.76±0.67	-4.44	0.001
		Gingival	15	1.17±0.54			
3	Group C	Occlusal	15	0.41±0.3	-0.66±0.48	-5.33	<0.001
		Gingival	15	1.07±0.4			

Comparing the microleakage scores at the occlusal and gingival bracket adhesive interface, there was a significant difference in microleakage scores at the occlusal and gingival bracket adhesive interfaces in Group A, Group B, and Group C (Table 2).

**Table 2:** Comparison of occlusal bracket adhesive and gingival bracket adhesive interface The level of significance is P <0.05

Sl. No	Groups	Bracket adhesive interface	N	Mean ± SD	Mean difference ± SD	t value	P-value
1	Group A	Occlusal	15	0.34±0.29	-0.85±0.59	-5.56	<0.001
		Gingival	15	1.19±0.6			
2	Group B	Occlusal	15	0.76±0.5	-0.87±0.59	-5.65	<0.001
		Gingival	15	1.63±0.35			
3	Group C	Occlusal	15	0.67±0.51	-0.76±0.67	-4.41	0.001
		Gingival	15	1.43±0.55			

Intergroup comparison of microleakage scores at the occlusal tooth adhesive interface, occlusal bracket adhesive interface, gingival tooth adhesive, and gingival bracket adhesive interface demonstrated that all the groups showed higher values of microleakage scores for nanocomposites followed by nanoionomers and Transbond XT showing the least (Table 3).

**Table 3:** Comparison of three groups on all four interfaces (occlusal tooth adhesive, occlusal bracket adhesive, gingival tooth adhesive, and gingival bracket adhesive interface) The level of significance is P <0.05 ANOVA: analysis of variance

Interfaces	Group A (n=15)	Group B (n=15)	Group C (n=15)	ONE-WAY ANOVA		POST-HOC TUKEY TEST		
				F value (*=Welch test)	P-value	Group A vs Group B difference (p-value)	Group A vs Group C difference (p-value)	Group B vs Group C difference (p-value)
Occlusal Tooth Adhesive interface	0.19±0.38	0.41±0.41	0.41±0.3	1.755	0.185	-0.22 (0.244)	-0.22 (0.251)	0 (1)
Occlusal Bracket Adhesive interface	0.34±0.29	0.76±0.5	0.67±0.51	4.855*	0.016	-0.41 (0.037)	-0.33 (0.122)	0.09 (0.848)
Gingival Tooth Adhesive interface	0.43±0.7	1.17±0.54	1.07±0.4	7.752	0.001	-0.74 (0.002)	-0.64 (0.009)	0.1 (0.872)
Gingival Bracket Adhesive interface	1.19±0.6	1.63±0.35	1.43±0.55	2.699	0.079	-0.43 (0.064)	-0.24 (0.415)	0.19 (0.556)

However, ANOVA revealed that the difference was statistically insignificant at occlusal tooth adhesive and gingival bracket adhesive interface. Post-hoc Tukey test depicted a statistically significant difference in microleakage scores between 1) Group B and Group C at the occlusal bracket adhesive interface and gingival tooth adhesive interface, and 2) Group A and Group C at the gingival tooth adhesive interface. ANOVA showed a significant difference between the mean microleakage scores among the groups but not between Group B and Group C (Table 4).

**Table 4:** Intergroup comparison of mean microleakage The level of significance is P <0.05

Intergroup	P values
Group A and Group B	0.000 (significant)
Group A and Group C	0.003 (significant)
Group B and Group C	0.616 (non-significant)

## Discussion

The problems associated with microleakage have been studied over the years, amongst which the prevention/development/management of incipient carious lesions (white spots) was given paramount importance. Microleakage has a role to play in the debonding of brackets during treatment, thereby making it a vital aspect to be analyzed and evaluated.

It is a consensus that the type of bonding cement used in bracket bonding has a direct influence on microleakage [11]. The degree of leakage is also determined by the composition of adhesive cement. Hence, it is of prime importance to identify those adhesive materials that exhibit optimal adhesiveness with minimal microleakage.

Although in literature, microleakage experiments concerning conventional adhesives and nanocomposites are plenteous, in-vitro studies associating the assessment of microleakage associated with nanoionomers and its comparison with other adhesive systems, are relatively less. Therefore, the present study aimed to evaluate and compare microleakage under orthodontic brackets bonded with the conventional adhesive system, nanocomposites, and nanoionomers.

Since their inception, nano-filled composites have proven to be a forerunner in restorative and/or esthetic dentistry. The vast surface area of traditional fillers makes it cumbersome for filler particles to penetrate resin tags following acid etching, thus portraying resin tags as filler-free zones. Therefore, with the development of nanotechnology, the aforesaid constraint may be overcome, as the filler particles can now be incorporated into resin tags, thereby having supplementary benefits, such as increased strength, reduced thermal expansion, reduced polymerization shrinkage, and improved bonding efficiency [12].

One of the most notable advancements in the field of adhesive dentistry has been the introduction of nanoionomers, wherein resin-modified glass ionomer cement (RMGIC) was manufactured solely based on bonded nanofiller technology. RMGICs have added advantage of their anti-cariogenic and remineralizing capabilities due to significant local fluoride reactions [13]. Ketac N100 is one such light-curing nanoionomer composed of fluoroaluminosilicate glass and nanofillers, presenting itself with enhanced mechanical properties and high fluoride release, replenishable following exposure to a topical fluoride source [14]. In addition, in vitro investigations, revealed that Ketac N100 can establish a caries inhibitory zone following acid exposure [9]. As a result, nanoionomers are a viable choice for an orthodontic adhesive in circumstances when the patient's oral hygiene is inadequate.

On ingestion of hot or cold meals, varying amounts of expansion and contraction of the teeth are perceived. These rates will differ for the teeth and the material if the latter's coefficient of thermal expansion does not match with the former. Due to the aforementioned effect, fluids are drawn in and pushed out at the borders of a restoration. This phenomenon is termed percolation [15]. Thermocycling and aging techniques were used in this study to simulate this percolation, as the study intended to analyze microleakage scores in the mouth after a while, thereby simulating the thermal changes in the oral cavity, fostering successive thermal stresses at the tooth-resin interface.

According to the results produced in our study, microleakage scores at the enamel-adhesive (E-A) and adhesive-bracket (A-B) interfaces were greater on the gingival side than on the occlusal side for all groups. This was in accordance with the results of various in vitro studies, wherein the gingival side of interfaces depicted higher microleakage than the occlusal side [10,14,16-19]. On comparing, the gingival side across all groups, i.e., Groups A, B, and C, demonstrated statistically significant microleakage scores at the E-A and A-B interfaces than occlusal sides. For the E-A interface, the brackets bonded with Transbond XT exhibited higher microleakage scores at the gingival side but were statistically insignificant. Variations in the gingival and occlusal scores can be attributed to the surface curvature anatomy, causing more adhesive material to be present on the gingival side according to Arhun et al. [15]. Ramoglu et al. anticipated that brackets would be drawn closer to the teeth due to the hardening of adhesive material near the light source caused by polymerization thereby, emanating an increased microleakage score of the adhesive farther away from the light source, due to the altered shrinkage characteristics [10].

The primary constituent of enamel is a homogenous arrangement of hydroxyapatite crystal prism bundles, which is further subdivided into an inner prismatic layer and an outer aprismatic layer. The outer prismless layer has an axis slightly parallel to the enamel surface, whereas the enamel rods of the inner prismatic layer are oriented perpendicular to the surface. One of the major goals of an etching process is to expose the inner enamel rods by erasing the outer aprismatic surface and if it fails to do so, it will then lead to a poorly etched surface of lower quality.

According to a scanning electron microscopy (SEM) study, on evaluating the etched surface of teeth, it was revealed that the middle and incisal regions exhibited a superior etch quality as compared to the cervical region, which was of poorer etch quality [20]. This was probably attributed to the findings that the incisal and middle-thirds of evaluated teeth demonstrated a prismatic honeycomb-like appearance as opposed to the aprismatic (prismless) surface of the cervical areas. The above can be justified by the fact that the enamel rods in the aprismatic cervical aspect of teeth are oriented in a parallel rather than perpendicular direction, toward the enamel surface, which again in all likelihood could also describe orthodontic bracket debonds. In most clinical scenarios, owing to the shorter height of the crown, orthodontic brackets are likely to be bonded onto the cervical aspect of both premolars and molars. As described in the previous paragraph, the etch quality is lower in the cervical area as compared to the middle and incisal thirds of etched teeth. In comparison, the enamel in the cervical region is thinner as opposed to the incisal and middle regions. The above inference is of paramount importance at a clinical level, as thinner enamel may render the region more prone to demineralization predisposing to white spot lesions around the gingival third of the teeth [21].

The amount of food debris and dental plaque accumulated on the gingival aspect of brackets is comparatively more in an in-vivo condition due to difficulty in cleaning these sites, which can even lead to periodontal issues, and secondary caries attributing to increased microleakage at the cervical interfaces [22].

On intergroup comparison, Group B (nanocomposites) evinced higher microleakage followed by Group C (nanoionomers) and Group A (Transbond XT) the least. A statistically significant difference was observed in the amounts of microleakage between the three groups in the occlusal A-B interface and gingival E-A interface. Our results were in accordance with other in vitro studies regarding the increased microleakage of teeth bonded with nanocomposites [6,23]. The reason for this increased microleakage, according to certain authors, can be linked to increased filler loading due to the reduced filler particle sizes leading to an increased surface-to-volume ratio. Hence, the water absorbency is increased, though increased filler particles improve the physical characteristics [24,25]. Low-viscosity composites have been used in several studies to increase adaptability and minimize microleakage [26,27]. As a result, the nanocomposite employed in this study had more microleakage than Transbond XT, which can be attributed to its more viscous nature. Nanoionomers, on the other hand, are less viscous, and hold an edge over nanocomposites, on account of their inherent property of being anti-cariogenic. However, there was no significant difference in the microleakage scores between Group B and Group C on intergroup comparison of mean microleakage scores.

Nanocomposites had a higher filler percentage by weight than nanoionomers as stated by Nayak et al. [28]. The chemical adhesion of the nanoionomer to the enamel surface, in addition to the polymerization by light cure, may have resulted in lower microleakage scores in the nanoionomer group as compared to the nanocomposite group. Due to the increased packable consistency of nanocomposite, the material penetrating the bracket mesh pads will be reduced, thereby surpassing the possible increase in microleakage expected due to moisture sensitivity in nanoionomers.

On another note, although the remineralization potential of nanoionomers is complemented by the release of large amounts of fluoride ions, they are, however, replenishable following exposure to a topical fluoride source, as stated by the manufacturers. All things considered, Ketac N 100 may be a superior choice for usage as an orthodontic adhesive under metallic brackets.

The null hypothesis was rejected for this study. On intergroup examination, there is a significant difference in the parameters between the groups of occlusal bracket-adhesive interface and gingival tooth-adhesive interface. There was no significant difference between nanoionomers and nanocomposites on the intergroup comparison of mean microleakage scores. On intragroup examination, the parameters showed a significant difference between the occlusal bracket adhesive interface and the gingival bracket adhesive interface in Groups A, B, and C. There was a significant difference between the occlusal tooth-adhesive interface and gingival tooth adhesive interface in Group B and Group C.

As a limitation, the application of primer on the base of the bracket could have improved the flowability of the materials employed in the study and might have caused decreased microleakage values. The pressure applied on the brackets for bonding it onto the tooth was not standardized, hence may create variation in values. The application of nanoionomers is a time-consuming and technique-sensitive procedure. Results and reasonings of an in vitro study to be precisely interpolated to the real world, are quite impossible.

## Conclusions

White spot lesions (WSLs) can be called an orthodontic cicatrix. One of the major reasons for the formation of WSL beneath the brackets would be microleakage, which crops up between the tooth adhesive. Microleakage also has a role in early bracket debonding, as there would be an ingress of oral fluids in the bracket adhesive interface. Within the limitations of the study design, occlusal sides in all groups exhibited lower microleakage scores compared with those observed in cervical sides for enamel-adhesive and adhesive-bracket interfaces. The intergroup comparison revealed significantly higher microleakage values for nanocomposites followed by nanoionomers and Transbond XT, the least.
